# Analyses of Avascular Mutants Reveal Unique Transcriptomic Signature of Non-conventional Endothelial Cells

**DOI:** 10.3389/fcell.2020.589717

**Published:** 2020-11-23

**Authors:** Boryeong Pak, Christopher E. Schmitt, Woosoung Choi, Jun-Dae Kim, Orjin Han, Jessica Alsiö, Da-Woon Jung, Darren R. Williams, Wouter Coppieters, Didier Y. R. Stainier, Suk-Won Jin

**Affiliations:** ^1^School of Life Sciences, Gwangju Institute of Science and Technology, Gwangju, South Korea; ^2^Curriculum in Genetics and Molecular Biology, University of North Carolina at Chapel Hill, Chapel Hill, NC, United States; ^3^Yale Cardiovascular Research Center and Section of Cardiovascular Medicine, Department of Internal Medicine, Yale University School of Medicine, New Haven, CT, United States; ^4^Department of Cardiovascular Sciences, Center for Cardiovascular Regeneration, Houston Methodist Research Institute, Houston, TX, United States; ^5^Unit of Animal Genomics, Faculty of Veterinary Medicine, Interdisciplinary Institute of Applied Genomics (GIGA-R), University of Liège (B34), Liège, Belgium; ^6^Department of Developmental Genetics, Max Planck Institute for Heart and Lung Research, Bad Nauheim, Germany

**Keywords:** zebrafish, endothelium, tailbud, fate mapping, transcriptomics

## Abstract

Endothelial cells appear to emerge from diverse progenitors. However, to which extent their developmental origin contributes to define their cellular and molecular characteristics remains largely unknown. Here, we report that a subset of endothelial cells that emerge from the tailbud possess unique molecular characteristics that set them apart from stereotypical lateral plate mesoderm (LPM)-derived endothelial cells. Lineage tracing shows that these tailbud-derived endothelial cells arise at mid-somitogenesis stages, and surprisingly do not require Npas4l or Etsrp function, indicating that they have distinct spatiotemporal origins and are regulated by distinct molecular mechanisms. Microarray and single cell RNA-seq analyses reveal that somitogenesis- and neurogenesis-associated transcripts are over-represented in these tailbud-derived endothelial cells, suggesting that they possess a unique transcriptomic signature. Taken together, our results further reveal the diversity of endothelial cells with respect to their developmental origin and molecular properties, and provide compelling evidence that the molecular characteristics of endothelial cells may reflect their distinct developmental history.

## Introduction

Endothelial cells (ECs) constitute the innermost lining of the vasculature and serve as an important therapeutic target for various diseases (Hogan and Schulte-Merker, 2017). It has been shown that the differentiation of ECs during development is regulated by the successive activation of transcription factors. For instance, in zebrafish, Npas4l, which belongs to the bHLH-PAS transcription factor family, appears to be at the top of the hierarchy and therefore functions as the master regulator (Reischauer et al., 2016). Npas4l has recently been shown to recognize the consensus sequence TCGTGA in promoter regions to regulate key endothelial transcription factor genes including *lmo2*, *tal1*, *etsrp*, as well as the extracellular matrix gene *egfl7* (Marass et al., 2019). The phenotype of *cloche* mutants, which is caused by a deleterious mutation in the *npas4l* locus, including lack of both endothelial and hematopoietic lineages, in agreement with the proposed role of Npas4l for the endothelial program (Stainier et al., 1995). Npas4l, in conjunction with Foxc1a/b, promotes the expression of *etsrp*, which encodes a member of the ETS domain transcription factor family, within presumptive endothelial progenitors (Veldman and Lin, 2012; Marass et al., 2019). Within the endothelial lineage, Etsrp functions as an essential regulator and its inactivation leads to a severe reduction of ECs in zebrafish (Sumanas et al., 2005; Sumanas and Lin, 2006). The severity of *etsrp* mutations, however, appears to be milder than that of *npas4l* mutations, since other members of the ETS domain transcription factors, including Fli1b, Erg, Etv4, Ets1, and Ets2 can partially compensate for the loss of *etsrp* (Pham et al., 2007; Liu and Patient, 2008; Liu et al., 2008; Craig et al., 2015). Consistent with their function, the expression of *npas4l* and *etsrp* is largely restricted to the lateral plate mesoderm (LPM) where the majority of ECs originate from Sumanas and Lin (2006), Reischauer et al. (2016).

Upon differentiation, ECs acquire common characteristics such as the expression of the lineage-specific markers *vegfr2*/*kdrl* and *cdh5* (Fang and Hirschi, 2019). However, increasing evidence suggests that ECs are a highly heterogeneous group of cells; it has been shown that different subtypes of ECs express unique sets of genes which define “subtype-specific” characteristics such as tip/stalk cells or arterial/venous ECs (Aird, 2007, 2012; Potente and Makinen, 2017). In addition, depending on the anatomical location they reside in, ECs are known to undergo differentiation to adopt organ- and/or tissue-specificity through reciprocal interaction with their surroundings, increasing the heterogeneity within the group (Red-Horse et al., 2007; Dejana et al., 2017). The complexity of ECs as a group has been confirmed by recent single cell transcriptomics, which revealed the extent of endothelial heterogeneity at the single cell level (Vanlandewijck et al., 2018; Kalluri et al., 2019; Dumas et al., 2020).

Not only are ECs diverse in terms of their cellular and molecular properties, but they also appear to have various developmental origins. A number of distinct regions within the embryo, generally mesodermal in nature, have been shown to harbor endothelial progenitors (Azar and Eyal-Giladi, 1979; Yablonka-Reuveni, 1989; Couly et al., 1995; Pouget et al., 2006; Stone and Stainier, 2019). While the majority of ECs are descendants of the progenitors within the LPM, which originated from the ventral margin of the gastrula (Melby et al., 1996; Shalaby et al., 1997; Vogeli et al., 2006; Warga et al., 2009; Prummel et al., 2019), other tissues including the somites, cephalic mesoderm, and tailbud can serve as additional sources for ECs to accommodate the rapid expansion of the embryo during somitogenesis (Couly et al., 1995; Wilting et al., 1995; Pouget et al., 2008; Martin and Kimelman, 2012; Nguyen et al., 2014; Row et al., 2016). For instance, it has been shown that the somite harbors undifferentiated bipotential progenitors that can generate ECs under *meox1* regulation (Nguyen et al., 2014). In addition, the posteriormost region of the embryo, the tailbud, contains posterior wall progenitor cells (PWPCs; also known as neuromesodermal progenitors) which can contribute to ECs (Row et al., 2016). Moreover, other germ layers could generate specialized ECs in specific circumstances. For instance, it has been reported that in mice, FOXA2^+^ endoderm-derived hepatoblast progenitors can contribute to ECs in the liver (Goldman et al., 2014). In pathological conditions, mesenchymal stem cells and cancer stem cells are also capable of generating disease-associated ECs in a context-dependent manner (Silva et al., 2005; Bussolati et al., 2009). Whether ECs from a non-conventional source possess unique cellular and molecular properties, which are distinct from ECs with a stereotypic origin, awaits further investigation.

Here, we report that a subset of ECs which does not require Npas4l or Etsrp for differentiation exists in zebrafish embryos. Using avascular mutants that lack either one of these two key transcription factors, we demonstrate that endothelial progenitors within the tailbud can give rise to ECs even in the absence of Npas4l or Etsrp. Compared to stereotypic ECs which are derived from the LPM, tailbud-derived ECs have a distinctive transcriptomic profile; in both microarray and single cell RNA-seq analyses, we find that the transcriptome of tailbud-derived ECs contains a significant percentage of somitogenesis- and neurogenesis-associated transcripts, which have not been previously implicated in the endothelial lineage. Taken together, our data suggest that the developmental origin of ECs may determine their molecular characteristics, which helps to create functional diversity.

## Materials and Methods

### Zebrafish Husbandry

All zebrafish (*Danio rerio*) were maintained under standard conditions in accordance with institutional and national guidelines, approved by the Institutional Animal Care and Use Committee. The following transgenic and mutants were used for the study: *Tg(kdrl:EGFP)*^*s*843^ (Jin et al., 2005), *clo*/*npas4l*^*s*5^ (Stainier et al., 1995), *etsrp*^*s*635^ (this manuscript), *etsrp*^*y*11^ (Pham et al., 2007), *cas*/*sox32*^*s*4^ (Alexander and Stainier, 1999; Alexander et al., 1999; Kikuchi et al., 2001), and AB wildtype. A new allele of *etsrp*, *s635*, was isolated from a previous large-scale forward genetic screen (Jin et al., 2007).

### Lineage Tracing

Lineage tracing was performed as previously described (Vogeli et al., 2006). Zebrafish embryos were injected with 0.2% w/v (2 μl of stock in 10 μl injection mix) DMNB-caged, biotinylated, lysine-fixable fluorescein dextran (10 kDa; Molecular Probes) in 0.2 M KCl with phenol red and HEPES at a 4.6 nL volume at one to two cell stage. For shield stage (6 h post-fertilization; hpf) uncaging, embryos were mounted laterally in 3% methylcellulose in 30% Danieau on a glass bottom dish. The 735 nm of a two-photon laser on a Zeiss 710 scope was used as a light source. The bleach function was set to 30 pulses. Embryos were examined to ensure the uncaging of a single cell, and those with supernumerary labeled cells were excluded for further analyses. The position of the cell was digitally recorded to trace the position of uncaged cells within the gastrula. For uncaging at 14 hpf, embryos were mounted on a glass-bottom dish in 1% low-melt agarose and exposed to UV light using the scan function of the DAPI channel on a Leica SP5. Region of interests were selected to specifically uncage the posterior somite or the tailbud. The embryos were collected and fixed for further observation at 26 hpf. Subsequently, sectioning and staining were performed as previously described (Vogeli et al., 2006). Following antibodies were used: chick anti-GFP (AB-CAM), goat anti-fluorescein (Invitrogen), donkey anti-goat 594 (Jackson Immunoresearch), donkey anti-chick 488 (Jackson Immunoresearch).

### Microarray and Single Cell Transcriptomics Analysis

To examine the expression pattern of the transcriptomes at developmental stage corresponding to our view, the previously published single cell RNA-seq (scRNA-seq) dataset (GSE112294) (Wagner et al., 2018) was re-analyzed using the Seurat package (v. 3.1.1) (Butler et al., 2018). We sought to visualize diverse subtypes of ECs during development. First, variable genes able to classify endothelial subtypes were identified based on variance stabilizing transformation (vst) using a normalized matrix. Next, principal component analysis (PCA) was performed followed by scaling scRNA-seq data. Subsequently, a plot graph was constructed by shared nearest neighbor (SNN) based clusters of the subtypes, and transformed to Uniform Manifold Approximation and Projection (UMAP) for dimension reduction for intuitive visualization.

To determine the developmental link between tailbud and *kdrl:*eGFP^+^ cells in avascular mutants, GFP^+^ cells from wildtype, *npas4l^–/–^, etsrp^–/–^*, and *sox32^–/–^* embryos in *Tg(kdrl:EGFP)*^*s*843^ transgenic background between 18 and 18.5 hpf were isolated via florescent activated cell sorting (FACS). RNA was extracted from the isolated cells using TRIzol and cDNA was synthesized using Maxima First Strand cDNA Synthesis Kit. Transcriptomic data from the triplicate samples of each genotype was measured using Agilent Zebrafish Genome Array (4 × 44K Probes). The raw data was primarily analyzed by BECMAN COULTER-Life Sciences Genomics. 11,036 intensity spots were selected after *P*-value test (*P* < 0.5) among identifiable 11,849 spots of total 37,598. The microarray data of each sample was normalized by quantile normalization method, and the normalized intensity spots were annotated according to the latest version from the database offered by NCBI, Ensemble, and ZFIN. To reduce analytic noise, the intensities of unannotated genes and internal control were eliminated for further analysis, consequently resulting in a total of 9,713 genes.

### Quantitative Analysis of Transcriptome

To avoid over-interpretation of single gene variation, often leading to a fault conclusion due to bias, we generated gene-set categories from 9,713 genes of the microarray data according to their functions (Supplementary Table 3). Given that *kdrl:*eGFP^+^ cells in avascular mutants emerge at the posteriomost region where somitogenesis or neurogenesis takes place, we hypothesized that the set of genes categorized by somitogenesis or neurogenesis could be utilized as indicators for non-conventional endothelial cells (ECs). Using those categories including “angiogenesis,” MVA-correlation coefficient was calculated among triplicates of each genetic sample following the “Pearson Correlation” formula. Subsequently, the coefficient values in the matrices were normalized upon the value of wildtype, leading to the scale of 0–1.

For further “non-biased” analysis to characterize molecular properties of *kdrl:*eGFP^+^ cells in avascular mutants, first, we generated the ‘quantile shift matrix’. The transcripts of the triplicate wildtype samples were ranked by the mean value of expressivity and divided into four quantiles (Q1–Q4); the first quantile contains the transcripts with lower expressions while the forth quantile contains those with higher expressions. The transcripts of *etsrp*^–/–^, and *npas4l*^–/–^ in each category—angiogenesis, somitogenesis, and neurogenesis—were placed according to the ranked WT transcripts, visualizing the variation in each quantile.

For in-depth analysis, the “modified histogram” in each category was generated. The ranked transcripts resulting from the “quantile shift matrix” were plotted by the correlation coefficients of each transcript set in *etsrp*^–/–^ or *npas4l*^–/–^ against that of WT. The correlation coefficients were normalized to the MVA-correlation coefficients of the whole transcriptome (Supplementary Figure 3D) to emphasize the significance of transcriptomic alterations over general differences of transcriptomic profiles among samples. The equation used for normalization is described below.

$$\begin{array}{rcl} & & \mathrm{Normalized correlation coefficient}\\ & & =\frac{\begin{array}{l} \mathrm{Correlation coefficient of ranked}\\ \mathrm{transcripts in each category} \end{array}}{\begin{array}{l} \mathrm{MVA}-\mathrm{correlation of whole transcriptome between}\\ \;\mathrm{avascular mutant and WT} \end{array}} \end{array}$$

### Gene Ontology (GO) Enrichment Analysis

To identify candidate genes potentially vital for non-conventional ECs, expression levels of transcripts were determined by luminous (fluorescent) intensity and transformed into log-ratio with the base 2. Differentially Expressed Gene (DEG) analysis was conducted between wildtype embryos and two avascular mutants to identify significantly up- or down-regulated genes. Those changed more than fourfold were considered as “significant” to minimize analytic noise. The functions of up or down-regulated genes were analyzed for ‘cellular component’ or ‘biological process’ using DAVID tools (Huang da et al., 2009a, b).

### Morpholino Injection, *in situ* Hybridization, and Quantitative Real-Time PCR

Morpholino injection and *in situ* hybridization were performed as previously described. The *npas4l* MO used in this study is 5′-CACCTGGAACACACAGTGGAGGATT-3′ (Reischauer et al., 2016). To determine the abundance of transcripts in *npas4l* MO-injected embryos, 24 hpf embryos were anesthetized by adding 25X tricane solution, and the trunk to tail regions were dissected and collected. Real-time PCR was performed using cDNA derived from the dissected samples. Gene expression was normalized to the housekeeping gene, *18S rRNA* and the endothelial specific gene, *kdrl*. Melting curve analysis was performed on all reactions. Data was analyzed using the 2^–ΔΔ*CT*^ method. Primers used in this study can be found in Supplementary Table 1.

### Statistical Analyses

Data presented in bar graphs present mean ± SD. For statistical analysis, GraphPad Prism software was used for data analysis. *P-*values were calculated using Unpaired Student’s *t*-test for two-group comparison. *p*-value (ns no significant change, ^∗^*p* < 0.05, ^∗∗^*p* < 0.005, ^∗∗∗^*p* < 0.0005, ^****^p < 0.00005).

## Results

### *s635* Is a Novel Allele of *etsrp*

From a previous large-scale forward genetic screen (Jin et al., 2007), we identified a novel avascular mutant, g*room of cloche* (*grc*^*s*635^). While morphologically indistinguishable from wildtype at early stages, expression of known endothelial markers such as *kdrl* and *flt4* were markedly reduced in *grc*^*s*635^ mutant, suggesting that they may possess a significantly reduced number of ECs (Supplementary Figure 1A). To better understand the molecular basis of this phenotype, we isolated the locus affected by the *grc*^*s*635^ mutation by whole genome sequencing and found a Leu248 to Pro248 change in *etsrp*, previously known as *etv2*, an essential transcription factor gene for ECs (Sumanas and Lin, 2006; Supplementary Figure 1B). Polyphen, a computational program which estimates the probability of damaging protein function and structure (Adzhubei et al., 2010), predicted that the amino acid substitution in *s635* is likely to be highly deleterious to the overall protein structure of Etsrp, suggesting that *s635* is likely to be a new loss of function allele of the *etsrp* gene (Supplementary Figure 1C). While the phenotype caused by the *s635* mutation appears to be stronger than the previously isolated *etsrp* null allele, *y11* (Pham et al., 2007), *s635* failed to complement *etsrp*^*y*11^ (Supplementary Figure 1D), indicating that *s635* is a novel loss-of-function allele of *etsrp*.

### Avascular Mutants Generate *kdrl*:eGFP^+^ Cells Later in Development

**FIGURE 1:**
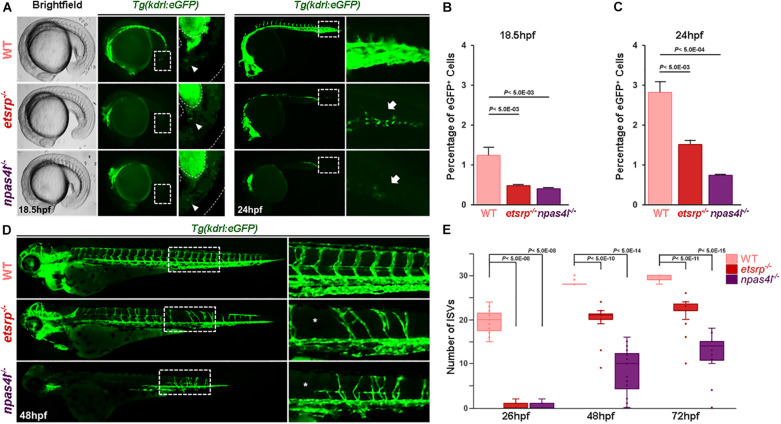
Avascular zebrafish generate *kdrl:*eGFP^+^ cells later in development. **(A)** Brightfield and fluorescence micrographs of 18.5 hpf (left two panels) and fluorescence micrograph of 24 hpf (right two panels) wildtype (top), *etsrp*^–/–^ (middle), and *npas4l*^–/–^ (bottom) in *Tg(kdrl:eGFP)* transgenic background. Areas within the white rectangles in 24 hpf images are shown in high magnification. Arrowheads (for 18.5 phf) and arrows (for 24 hpf) point the significant reduction of eGFP^+^ cells in the trunk of avascular mutants. **(B,C)** Quantification of the number of eGFP^+^ cells in 18.5 hpf **(B)** and in 24 hpf **(C)** wildtype, *etsrp*^–/–^, and *npas4l*^–/–^. Both avascular mutants have significantly fewer number of eGFP^+^ endothelial cells. **(D)** Fluorescence micrographs of 48 hpf wildtype (top), *etsrp*^–/–^ (middle), and *npas4l*^–/–^ (bottom) in *Tg(kdrl:eGFP)* transgenic background. Areas within the white rectangles are shown in high magnification. **(E)** Quantification of the number of intersegmental vessels in wildtype, *etsrp*^–/–^, and *npas4l*^–/–^ at 26, 48, and 72 hpf.

Since *etsrp*^*s*635^ mutants (henceforth *etsrp*^–/–^) display vascular defects reminiscent of a previously identified mutant, *cloche/npas4l*^*s*5^, (henceforth *npas4l*^–/–^), we compared the vasculature of these two mutants at distinct developmental stages using a transgenic line that selectively visualizes ECs, *Tg(kdrl:eGFP)*^*s*843^. As previously reported (Craig et al., 2015), eGFP^+^ cells, which are presumably endothelial in nature, begin to emerge at 18.5 hpf within the posteriormost region of *etsrp*^–/–^ embryos, despite the absence of ECs at earlier stages (Supplementary Movie 1 and Figures 1A,B). Similarly, a small number of eGFP^+^ cells was identified within the posteriormost region of *npas4l*^–/–^ (Figures 1A,B). In both mutants, the percentage of eGFP^+^ cells gradually increased, although it remained substantially lower than in wildtype at 24 hpf (Figures 1A,C). By 24 hpf, the eGFP^+^ cells in avascular mutants coalesced and generated a rudimentary vasculature with angiogenic sprouts toward the dorsal region of the embryos, of which number substantially increased by 48 hpf (Supplementary Movie 2 and Figures 1D,E). Considering that these cells express endothelial markers and display behavior reminiscent of ECs in wildtype embryos, our data suggest that progenitors which could give rise to ECs in the absence of Npas4l or Etv2 exist in these avascular mutants.

**FIGURE 2:**
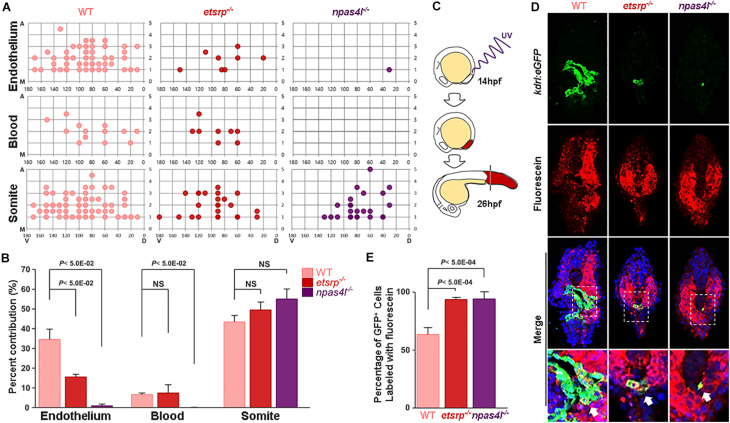
Specification of mesodermal fate is altered in avascular gastrulae. **(A)** Fate map with a single cell resolution at gastrulation stage was constructed by uncaging a single cell within the ventral margin of the gastrula and track their fate. Fate map within the ventral most five rows of gastrula are shown in detail. *X*- and *Y*-axes represent the dorsoventral and animal-vegetal axes, respectively. M in the *Y*-axis denotes margin of the gastrula, which is the vegetal-most region of the embryos. **(B)** In avascular mutants, the lineage distribution from the mesoderm appears to be altered; the percentage of the endothelium derived from the ventral margin of the gastrula was significantly reduced in both avascular mutants. In *npas4l*^–/–^ gastrula, the hematopoietic lineage was similarly affected. In contrast, the percentage of somite derived from the ventral margin of the gastrula was largely unaffected by either avascular mutant. **(C)** The posteriormost region of the embryo including posterior somites and the tailbud was illuminated with UV to uncage the caged fluorescein at 14 hpf, and the percentage of endothelial descendants from uncaged cells were assessed at 26 hpf. **(D)** Transverse section of 26 hpf wildtype (left), *etsrp*^–/–^ (middle), and *npas4l*^–/–^ (right) *Tg(kdrl:eGFP)* embryos stained with TOPRO (blue) and uncaged fluorescein (red). Areas within the white rectangles are shown in detail. White arrows point to the uncaged fluorescein*^+^*/eGFP*^+^* cells, which are presumably endothelial descendants of the uncaged cells. **(E)** Quantification of the percentage of fluorescein labeled endothelial cells within the endothelial population in the posteriormost part of the wildtype, *etsrp*^–/–^, or *npas4l*^–/–^ embryos.

To further determine the developmental origin of *kdrl*:eGFP^+^ in avascular mutants (henceforth non-conventional endothelial cells; ncECs), we constructed a single cell resolution fate map within the ventral margin of the gastrula which has been previously shown to possess endothelial progenitors (Warga and Nüsslein-Volhard, 1999; Vogeli et al., 2006; Warga et al., 2009), in 6 hpf wildtype, *etsrp*^–/–^ and *npas4l*^–/–^ (Figure 2A). In wildtype gastrulae, approximately 35% of the cells (out of 305 uncaged cells) within the ventral margin of the gastrula contributed to ECs at later stages, which was significantly reduced in avascular mutants (Figures 2A,B); the percentage of endothelial progenitors in the ventral margin of the gastrula was reduced to 15% in *etsrp*^–/–^, and less than 2% in *npas4l*^–/–^ (out of 195 and 141 uncaged cells in *etsrp*^–/–^ and *npas4l*^–/–^, respectively, Figure 2B). Therefore, it appears that *etsrp*^–/–^ retains a small group of ECs which comes from the progenitors originated from the ventral margin of the gastrula, while *npas4l*^–/–^ is likely to contain a negligible number of ECs derived from the ventral margin of the gastrula. Consistent with the previously reported phenotype (Stainier et al., 1995), the hematopoietic progenitors were also abrogated in *npas4l*^–/–^ in addition to the endothelial progenitors (Figures 2A,B). In both avascular mutants, we observed a slight yet persistent increase in the percentage of somitic cells, raising the possibility that the presumptive endothelial progenitors might differentiate as somites in the absence of Etsrp or Npas4l (Figure 2B). Considering that the ventral margin of the gastrula gives rise to the LPM at later stages (Prummel et al., 2019), our data suggest that a significant portion of ECs in avascular mutants are not likely to arise from the LPM.

### ECs in the Posterior Region of Avascular Mutants Emerge From the Tailbud

Given that ECs in avascular mutants were restricted to the posteriormost region of the embryo at 18.5 hpf, we hypothesized that ECs in avascular mutants originate from endothelial progenitors residing within the posteriormost somites or the tailbud region (Nguyen et al., 2014; Row et al., 2016). To directly test this possibility, we performed *in vivo* lineage tracing by using photo-convertible fluorescein deducing the endothelial contribution of two distinct regions corresponding to the posteriormost somites (8th, 9th, and 10th somite) and the tailbud in avascular mutants. Cells within the posterior region of the caged fluorescein-conjugated dextran injected wildtype or avascular mutants were uncaged at 10-somite stage (14 hpf), and the percentage of fluorescein-labeled *kdrl:*eGFP^+^ ECs located posterior to the yolk extension was quantified at 26 hpf when ECs emerge in both avascular mutants (Figure 2C). Subsequently, a series of transverse sections of the uncaged embryos were analyzed to deduce the contribution of local progenitors to ECs (Figure 2D). Both wildtype and avascular mutants possess fluorescein-labeled *kdrl:*eGFP^+^ population in the posterior part of the embryos, indicating that these cells are likely to be descendants of the posteriormost somites and the tailbud (Figure 2D). However, we were not able to formally exclude the possibility that the posterior LPM contributes to the eGFP^+^ cells in avascular mutants. Approximately 65% of wildtype ECs in the posterior region, and over 95% of avascular ECs in the posterior region were fluorescein^+^/eGFP^+^ (Figure 2E). Taken together, our data suggest that presumptive endothelial progenitors in the tailbud such as the PWPCs could serve as an important source for ECs in the posterior region. Moreover, it appears as if these progenitors undergo endothelial differentiation independently of Npas4l or Etsrp since their differentiation appears to be unaffected in avascular mutants.

### The Fate of Non-conventional ECs Might Be Determined by Alternative Mechanisms

**FIGURE 3:**
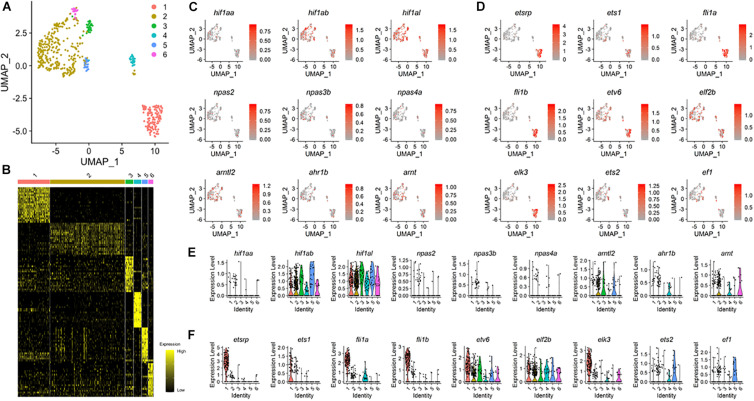
Expression pattern of bHLH-PAS and ETS genes in 18 hpf zebrafish ECs. **(A)** UMAP visualization from published data (GSE112294) reveals six distinct clusters of the *kdrl*^+^ endothelial cells in 18 hpf embryos. **(B)** Heat map shows expression of unique gene sets for each cluster defined in **(A)**, with yellow and black indicating higher and lower expression, respectively. **(C)** Expression pattern of known bHLH-PAS family members within clusters of endothelial cells. **(D)** Expression pattern of known ETS family members within clusters of endothelial cells. At least five ETS genes, *etv6*, *elf2b*, *elk3*, *ets2*, and *ef1* show distinct expression pattern than *etsrp*. **(E,F)** Violin plots of individual bHLH-PAS family **(E)** and ETS family **(F)** genes.

Our data show that ECs can arise from the region of the tailbud in the absence of either Npas4l or Etsrp, raising the possibility that differentiation of ECs from distinct progenitors is regulated by subtype-specific molecular mechanisms. To test this idea, we re-analyzed previously reported scRNA-seq data by isolating *kdrl*^+^ cells (Wagner et al., 2018), and identified six distinct clusters, each of which might represent a unique endothelial subtype within 18 hpf zebrafish (Figures 3A,B). Cluster 1 appears to strongly express well-characterized endothelial markers, including *etsrp*, *flt1*, and *flt4*, indicating that it represents conventional *kdrl*^+^/*etsrp*^+^ ECs which predominantly arise from the LPM during development (Supplementary Figures 2A,B). In addition, other clusters also contain *flt1* and *flt4* expressing cells, reflecting their endothelial identity (Supplementary Figures 2A,B). Considering that Npas4l and Etsrp belong to the bHLH-PAS family and the ETS transcription factor family respectively (Sumanas et al., 2005; Reischauer et al., 2016), other members of the family could promote the differentiation of ECs from non-conventional sources including the tailbud. To examine this possibility, we analyzed the expression pattern of the bHLH-PAS family and the ETS transcription family members among the six endothelial clusters.

UMAP visualization showed that transcripts of bHLH-PAS family genes were detected within *kdrl*^+^ cells. While we were not able to detect expression of *npas2*, *npas3b*, and *npas4a* (Figure 3C), distant paralogs of *npas4l* were broadly expressed in ECs at 18 hpf (Figures 3C,E), with *ahr1b* being the only bHLH-PAS family gene of which expression was restricted to a specific cluster (Figures 3C,E). Since *npas4l* has been recently identified and was not previously annotated in the genome (Reischauer et al., 2016), it is conceivable that npas4l was not included for the analyses. In 18 hpf *kdrl*^+^ ECs, a number of ETS domain transcription factor family genes were expressed with distinct distribution among the six clusters of ECs (Figures 3D,F). Three ETS domain transcription factor genes, *ets1*, *fli1a*, and *fli1b*, co-localized with *etsrp* and were restricted to cluster 1 (Figures 3D,F). On the contrary, five ETS genes, *etv6*, *elf2b*, *elk3*, *ets2*, and *ef1*, were strongly expressed in clusters 2–6 and displayed expression patterns distinct from *etsrp* (Figures 3D,F). Therefore, these factors may substitute for *etsrp* to promote endothelial differentiation in a subset of non-conventional *kdrl*^+^/*etsrp*^–^ ECs during development.

### Expression of Transcription Factors Are Preferentially Altered in Non-conventional ECs

Our data indicate that ECs are a heterogeneous population composed of distinct subpopulations derived from diverse origins. To better understand the unique molecular properties of ncECs, in particular the tailbud-derived ECs, we compared the transcriptome of wildtype and avascular mutants, since avascular mutants possess a higher proportion of non-conventional tailbud-derived ECs at 18 hpf, the earliest stage when *kdrl*:eGFP cells could be detected (Figure 1A and Supplementary Figures 3A,B). We included *cas*/*sox32*^*s*4^ (henceforth *sox32*^–/–^), which completely lacks endoderm but retains normal vasculature (Alexander and Stainier, 1999; Jin et al., 2005) to exclude eGFP^+^ endodermal cells from subsequent analyses (Supplementary Figure 3A). In 18 hpf wildtype, *kdrl:*eGFP^+^ cells constituted approximately 3% of total cells, while *etsrp*^–/–^ and *npas4l*^–/–^ contained a significantly lower number of *kdrl:*eGFP^+^ cells (Supplementary Figure 3C). Of note, *etsrp*^–/–^ had more *kdrl:*eGFP^+^ cells than *npas4l*^–/–^, reflecting the differences in the severity of the endothelial phenotype.

Multivariate analysis (MVA) of which value was scaled 0–1, showed more than 0.849 overall correlation among transcriptomes of the same genotype (Supplementary Figure 3D), which was recapitulated by Principle Component Analysis (PCA). Therefore, the differences in transcriptomic profiles of each sample appeared to be dependent on the genotypes (Supplementary Figures 3D,E). To exclude genes of which the expression was enriched in the pharyngeal endoderm and therefore were misinterpreted as “upregulated” in either avascular mutant, we compared the transcriptome of wildtype and *sox32*^–/–^ samples, and selected 7020 “non-pharyngeal endoderm” genes for further analyses (Supplementary Figure 3F).

Comparison of those 7,020 genes showed that 786 genes were upregulated and 1,037 genes were downregulated in *etsrp*^–/–^ compared with wildtype (Supplementary Figure 4A). In *npas4l*^–/–^, 918 genes and 1,475 genes were upregulated and downregulated, respectively (Supplementary Figure 4B). Next, we used Gene Ontology enrichment in terms of “cellular components” (GO: Cellular components) for further analysis on transcriptomic profiles of ncECs from avascular mutants. GO analysis showed pronounced changes in expression of transcription factors associated with developmental regulation in both avascular mutants (Supplementary Figures 4C,D), which are reminiscent of the results obtained from the re-analysis of single cell transcriptomics data (Figures 3C,D).

### Somitogenesis- and Neurogenesis-Associated Transcripts Are Enriched in Non-conventional ECs

**FIGURE 4:**
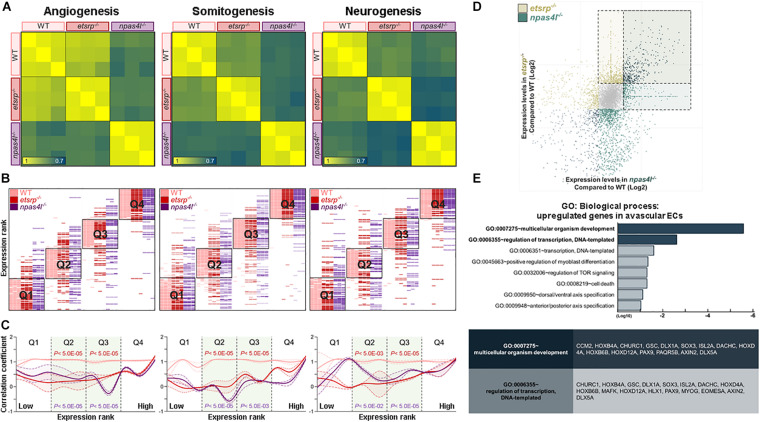
Transcriptome analyses reveal unique transcriptomes of avascular ECs. **(A)** Correlation matrices for angiogenesis- (left), somitogenesis- (middle), and neurogenesis-associated (right) transcripts. Correlation of expression level among wildtype, *etsrp*^–/–^ and *npas4l*^–/–^ endothelial cells is lower in somitogenesis- or neurogenesis-associated transcripts than in angiogenesis-associated transcripts. **(B)** Quantile shift analysis visualizes variations of angiogenesis- (left), somitogenesis- (middle), and neurogenesis-associated (right) transcripts among wildtype, *etsrp*^–/–^ and *npas4l*^–/–^ samples. **(C)** A modified histogram shows differences in correlation coefficient of wildtype, *etsrp*^–/–^, and *npas4l*^–/–^ endothelial cells depending on their expression ranks. In both avascular mutants, correlation coefficient of transcripts associated with somitogenesis (middle) or neurogenesis (right) is generally lower than that of transcripts associated with angiogenesis (left). The correlation coefficient of each sample was normalized to those of corresponding samples. **(D)** Expression of 931 transcripts was significantly altered in both *etsrp*^–/–^ and *npas4l*^–/–^ endothelial cells with 376 upregulated transcripts and 555 downregulated transcripts. **(E)** Gene Ontology enrichment analysis shows transcripts encoding developmentally regulated proteins enriched in endothelial cells from avascular mutants.

To further analyze the transcriptomic signature of ncECs isolated from the *etsrp*^–/–^ or *naps4l*^–/–^ mutants in a non-biased manner, we generated MVA-correlation matrices for angiogenesis-, somitogenesis-, and neurogenesis-associated transcripts from 7,020 genes (Supplementary Table 3). We chose transcripts within the somitogenesis or neurogenesis categories in addition to angiogenesis, since ECs derived from the tailbud are known to share progenitors with somites and neurons (Row et al., 2016). MVA-correlation matrices revealed that correlations of the transcripts belong to angiogenesis category were higher among WT, *etsrp*^–/–^, and *npas4l*^–/–^ than those of somitogenesis or neurogenesis categories (Figure 4A). This result implies that lack of either Etsrp or Npas4l appears to influence the expression of genes associated with somitogenesis and neurogenesis in ECs wildtype.

To assess the differences in the transcriptomic profiles among wildtype, *etsrp*^–/–^, and *npas4l*^–/–^, we further examined the alteration of transcriptomic profiles in avascular mutants. All transcripts in each category were ordered according to their expression in wildtype, and divided into four quantiles Q1–Q4. Then, the rank of each transcript within a specific quartile was comparedbetween WT and avascular mutants. In all three categories, the rank of the transcripts in Q2 and Q3 tend to fluctuate more compared to the rank of those in Q1 or Q4 (Figure 4B). Therefore, these rank shifts of the transcripts in Q2 and Q3 could visualize the differences in transcriptomic profiles among the three biological samples. Next, we sought to reveal the significance of the transcriptomic changes in ncECs within each category. Therefore, we designed a “modified histogram” of categories upon their expression ranks plotted against the normalized correlation coefficient (see section “Materials and Methods”). The overall correlation coefficient of Q2 and Q3 in avascular mutants was higher in angiogenesis-associated transcripts while lower in somitogenesis- or neurogenesis-associated transcripts (Figure 4C). Taken together, our data suggest that the transcriptomic profiles of ncECs are likely to contain unique characteristics.

### Non-conventional Tailbud-Derived ECs Possess Unique Molecular Characteristics

To further define unique molecular characteristics of ncECs, we selected 921 transcripts whose expression was altered in both *etsrp*^–/–^ ECs and *npas4l*^–/–^ ECs with 376 upregulated and 555 downregulated transcripts when compared to wildtype (Figure 4D and Supplementary Figure 4E). We postulated that these 376 transcripts may reflect the transcripts specific to ncECs which are derived from the tailbud, because they are likely over-represented in both avascular mutants (Supplementary Table 2). Among these, the number of transcripts associated with somitogenesis or neurogenesis were more abundant than those associated with angiogenesis (Supplementary Figures 4E,F). Consistent with our analysis, the expression of these transcripts was elevated in *npas4l* MO-injected embryos (Supplementary Figures 5A,B).

GO enrichment analysis (GO: Biological process) of these 376 up-regulated transcripts in both *etsrp*^–/–^ and *npas4l*^–/–^ ECs revealed that the majority of the transcripts are associated with multicellular organism development, regulation of transcription, and dorsal/ventral patterning (Figure 4E). Among the upregulated transcripts, a number of genes known to be expressed in the tailbud were identified, including *isl2a*, *hoxd12a*, *hoxb4a*, *dlx5a*, *hoxb6b*, *axin2*, *sox3*, *dact1*, and *hoxd4a*. Taken together, our data suggest that transcripts enriched in both avascular mutants may reflect molecular characteristics of ncECs, in particular, the tailbud-derived ECs.

**FIGURE 5:**
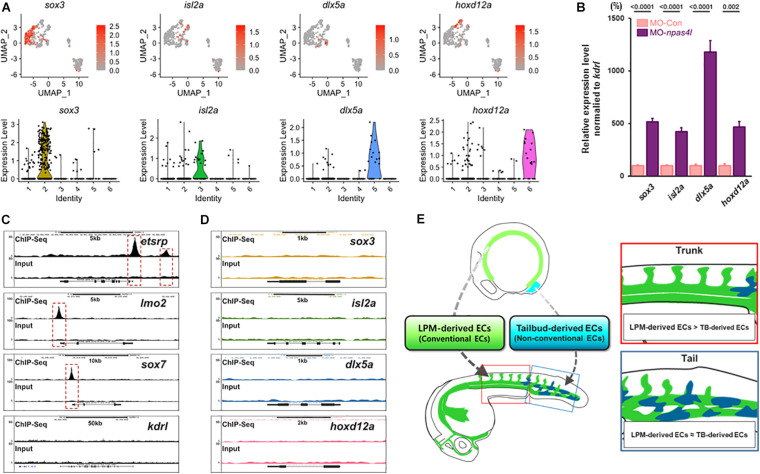
scRNA-seq analysis reveals distinct subpopulation of ECs. **(A)** Distribution of transcripts of which expression is enriched in *kdrl*^+^/*etsrp*^–^ clusters are shown (top) with corresponding Violin plot (bottom). Representative transcripts for each *kdrl*^+^/*etsrp*^–^ cluster (*sox3* for cluster 2, *isl2a* for cluster 3, *dlx5a* for cluster 5, and *hoxd12a* for cluster 6) are shown in detail. **(B)** Quantitative Real time PCR (qRT-PCR) on samples obtained from the posterior region of 24 hpf *npas4l* MO-injected embryos shows elevated expression of aforementioned *kdrl*^+^/*etsrp*^–^ cluster-enriched transcripts. **(C,D)** *in silico* re-analysis of ChIP-seq of canonical transcripts in endothelial cells. **(C)** Npas4l binding sites are present in the proximal regulatory region of endothelial genes including *etsrp* and *lmo2*. Of note, *kdrl* does not contain any obvious Npas4l binding sites in the proximal regulatory region. **(D)** In contrast, consensus Npas4l binding sites are absent in the proximal regulatory region of genes whose expression is enriched in *kdrl*^+^/*etsrp*^–^ clusters. **(E)** Our proposed model: developing zebrafish embryos are likely to contain endothelial cells that originate from the lateral plate mesoderm (green colored) as well as non-conventional sources such as the tailbud (blue colored). While the endothelial cells from non-conventional sources are rare within the endothelial population, we speculate that the proportion of these cells is enriched in the posterior region of the embryo.

We selected *sox3*, *isl2a*, *dlx5a*, and *hoxd12a* for further analysis, which could potentially serve as selective markers for tailbud-derived ECs based on their enrichment in a specific cluster in single cell analysis (Supplementary Figure 5C), expressivity in avascular ECs, and their proposed biological function (Figure 5A and Supplementary Table 3). Expression of these transcripts were enriched in *kdrl*^+^/*etsrp*^–^ clusters, which seemed to contain ncECs. In *npas4l* MO-injected embryos, expression of these transcripts appeared to be elevated (Figure 5B). Re-analysis of previously reported genome-wide chromatin immunoprecipitation sequencing (ChIP-seq) data (Marass et al., 2019) revealed that these ncEC-associated transcripts lack Npas4l consensus binding sites in the proximal regulatory region, while known endothelial markers display presence of Npas4l binding motifs (Figures 5C,D). Therefore, it appears that Npas4l does not directly regulate the expression of these transcripts.

## Discussion

In this report, we find that avascular zebrafish mutants could generate *kdrl:*eGFP^+^ ECs, which predominantly emerge from the progenitors residing within the tailbud at later developmental stages. These ECs appear to represent a unique subpopulation; not only do these ECs have distinct developmental origins, but they also possess unique molecular characteristics. Using multimodal transcriptomic analyses, we identify that these tailbud-derived ECs in avascular mutants have elevated expression of transcription factors associated with somitogenesis or neurogenesis. Based on our finding, we propose that the endothelial population contains a number of subsets with distinct molecular characteristics and developmental history (Supplementary Figure 5C and Figure 5F). While the majority of ECs derive from the LPM, a significant fraction of ECs appears to emerge from other tissues such as the tailbud.

Our finding suggests that a subset of ECs could emerge from the tailbud region independent of Etsrp and Npas4l function, illustrating the complexity of endothelial differentiation *in vivo* (Figure 5E). Single cell transcriptomic analysis, which shows that a number of Npas4l and Etsrp orthologs are expressed in a subset of ECs and could partially compensate for the loss of these factors, further supports this idea. This is consistent with previous reports suggesting that *etsrp*^–/–^ and *npas4l*^–/–^ retain a small number of ECs (Liao et al., 1997; Martin and Kimelman, 2012; Craig et al., 2015). In *etsrp*^–/–^, pre-existing ECs have been implicated as the origin of ECs, however, it has not been fully elucidated where these pre-existing ECs arise from during development (Craig et al., 2015).

Our fate map shows that ECs in avascular mutants do not originate from the ventral margin of the gastrula, the conventional source for ECs (Melby et al., 1996; Vogeli et al., 2006), but from the tailbud of the embryos approximately at 14 hpf, although we cannot formally exclude the possibility that a small percentage of these cells might have a different developmental origin. Our finding is consistent with previous reports that the tailbud harbors progenitors capable of generating the entire non-epidermal posterior body including ECs (Martin and Kimelman, 2012; Row et al., 2016), and in agreement with a recent report that *npas4l*^–/–^ does not contain either LPM- or somite-derived ECs (Sahai-Hernandez et al., 2020). Considering that highly plastic progenitors, the PWPCs which are proposed to retain angiogenic potential, exist in the tailbud (Row et al., 2016), we speculate that these cells may give rise to ECs in avascular mutants.

Since our data indicated that the majority of ECs in avascular mutants originate from the tailbud, we were able to deduce unique molecular and cellular profiles of tailbud-derived ECs using avascular mutants as a tool. We show that tailbud-derived ECs could form rudimentary vasculature and undergo sprouting angiogenesis *in vivo* in the absence of conventional LPM-derived ECs, indicating that these two subtypes of ECs are likely to be functionally equivalent. At molecular level, in addition to conventional endothelial markers, tailbud-derived ECs show characteristic upregulation of tailbud-associated genes and retain expression of transcription factors which have been implicated in neuronal development or axis formation, reflecting their distinctive developmental origin.

Interestingly, the majority of transcripts of which expression was elevated in tailbud-derived ECs appears to be associated with either somitogenesis or neurogenesis rather than angiogenesis. Given that the presumptive endothelial progenitors in the tailbud, the PWPCs, also serve as a progenitor for both posterior somite and neurons (Row et al., 2016), our data suggest that the transcriptomic signature of the tailbud-derived ECs may reflect their distinct developmental history as suggested by recent reports (Chan et al., 2019; Weinreb et al., 2020). The majority of the transcripts of which expression was elevated in avascular ECs appears to be transcription factors, suggesting the possibility that these transcription factors may function as cell fate regulators in undifferentiated tailbud progenitors.

Since *kdrl:*eGFP^+^ cells emerge even in absence of Etsrp or Npas4l, which function at the top of the hierarchy regulating differentiation of ECs, we postulate that tailbud-derived ECs not only possess distinct molecular characteristics, but also are differentially regulated during specification. Lack of known Etsrp or Npas4l binding sites in the promoter of genes of which expression is selectively upregulated in ECs of avascular mutants further supports this idea. Therefore, we propose that yet unidentified transcription factors may replace the function of Etsrp and Npas4l and coordinate the differentiation of ECs from PWPCs in the tailbud. Since both Etsrp and Npas4l belong to a large group of transcription factor family, it is possible that one of their paralogs may take over the function of Etsrp and Npas4l in tailbud-derived ECs. For instance, our re-analysis on the previously published single cell transcriptomic dataset suggests that a number of ETS factors display distinct expression domains within *kdrl*^+^ cells, in particular *etv6*, *elf1*, *ets2*, and *elf2b*. It is interesting to note that *elf1* and *elf2b* have shown to be expressed independent of *npas4l* (Liu and Patient, 2008), and that MO-mediated inhibition of *ets2* did not cause significant vascular defects (Sumanas and Lin, 2006; Pham et al., 2007), further substantiating our idea that tailbud-derived ECs, which constitute only a small percentage of total ECs, may arise independent of Npas4l and Etsrp during development.

While our analyses revealed distinct molecular property of tailbud-derived ECs, detailed understanding of the functional importance of these cells *in vivo* warrants further investigation. Considering that the tailbud of mammals retains multipotent progenitors similar to PWPCs in zebrafish (Naruse-Nakajima et al., 2001; Tzouanacou et al., 2009), our analyses on tailbud-derived ECs could provide novel framework to understand how diverse developmental origins could influence the function of ECs in health and disease, and promote the development of therapeutic interventions for various human diseases with vascular components.

## Data Availability Statement

The datasets generated for this study can be found in EMBL accession E-MTAB-9698.

## Ethics Statement

The animal study was reviewed and approved by Gwangju Institute of Science and Technology LARC.

## Author Contributions

BP, CS, WCh, J-DK, OH, and JA performed the material preparation, data collection, and analysis. D-WJ, DW, WCo, and DS provided key reagents, S-WJ wrote the first draft of the manuscript. All authors commented on previous versions of the manuscript, read and approved the final manuscript, and contributed to the study conception and design.

## Conflict of Interest

The authors declare that the research was conducted in the absence of any commercial or financial relationships that could be construed as a potential conflict of interest.
