# Supplementary material for: Analyses of Avascular Mutants Reveal Unique Transcriptomic Signature of Non-conventional Endothelial Cells
Source: Front Cell Dev Biol. 2020 Nov 23;8:589717. doi: 10.3389/fcell.2020.589717 (PMC7719722; doi:10.3389/fcell.2020.589717)
Supplement: Supplementary file 1 [file Data_Sheet_1.docx]

**Supplementary Figures**

**
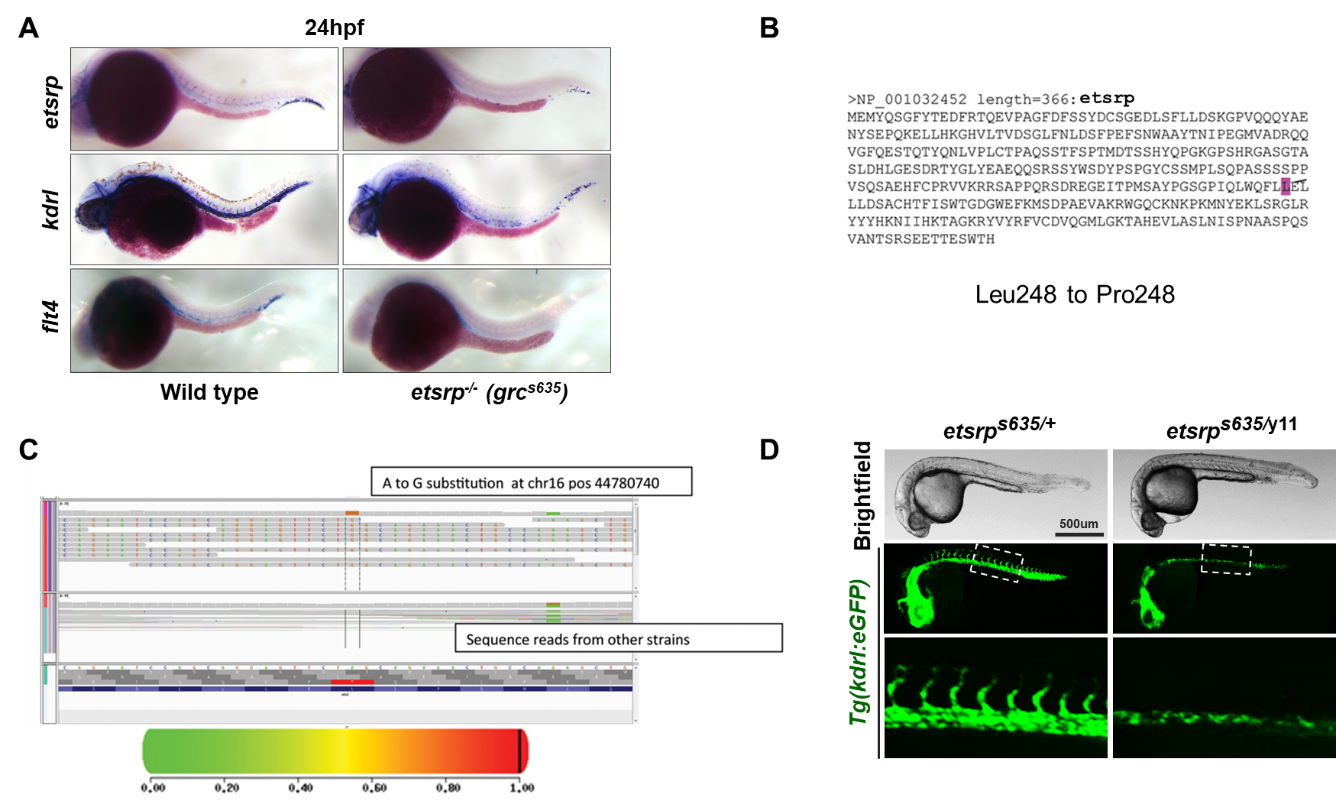
**

**Supplementary Figure 1: Isolation of *s635*, a novel allele of *etsrp***

(**A**) Expression of endothelial genes including *etsrp*, *kdrl*, and *flt4* is drastically reduced in *s635* embryo. (**B**) Whole genome sequencing identifies Leu248 to Pro248 change in *etsrp* locus. (**C**) Polyphen predicts that a change from Leu248 to Pro248 could be highly damaging to the overall protein structure. (**D**) Brightfield (top) and fluorescence (bottom) images of 30hpf *etsrp^s635/+^* (left) and *etsrp^s635/y11^* (right) embryos. Areas within the rectangles in the middle panels are shown in high magnification in the bottom panels.


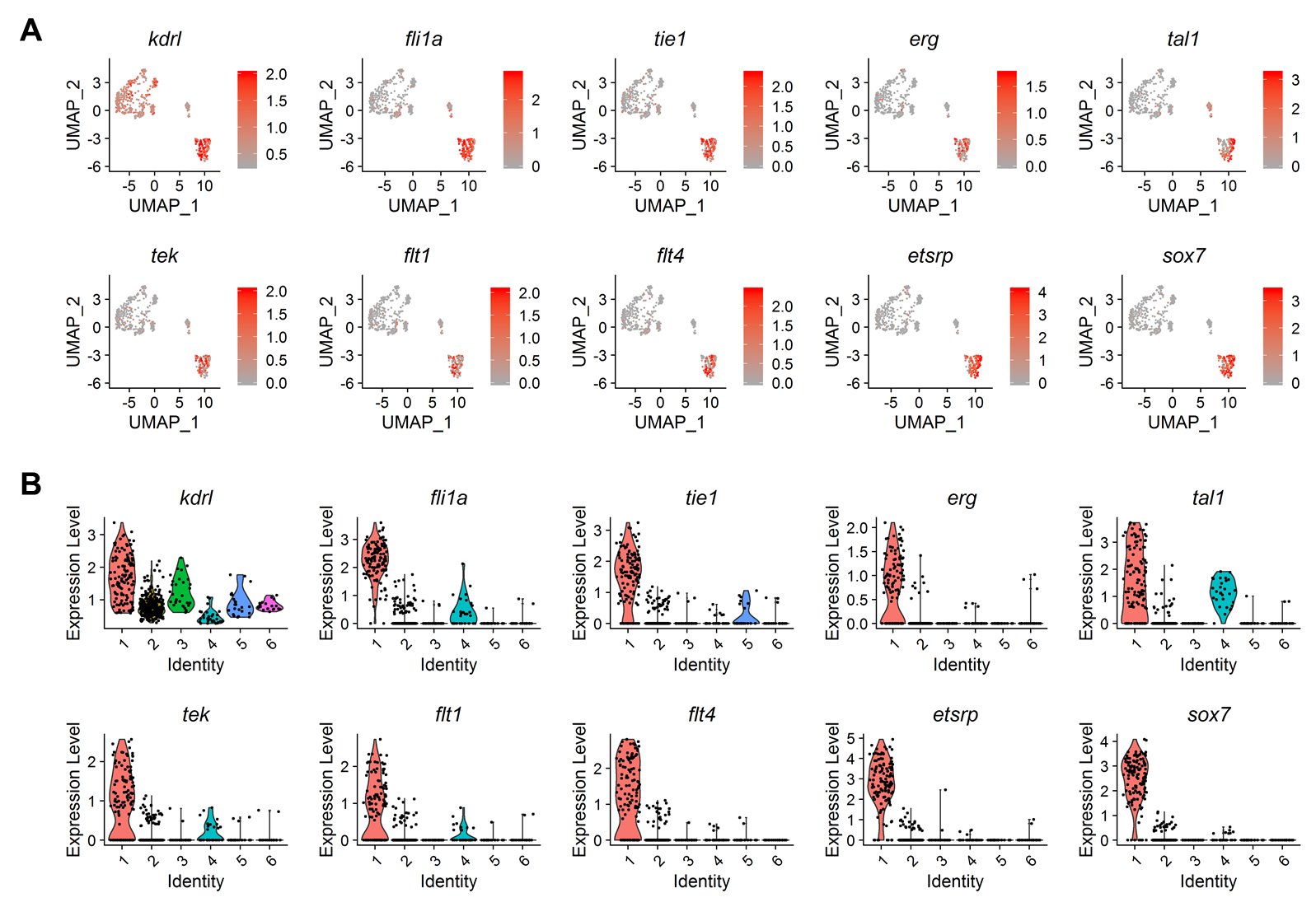


**Supplementary Figure 2: UMAP visualization of endothelial genes expression in zebrafish**

(**A**) UMAP visualization reveals distinct expression domains of canonical endothelial markers in endothelial cells isolated from 18hpf zebrafish embryo. The majority of endothelial markers is preferentially expressed in cluster 1 and co-localized with *etsrp*. (**B**) Violin plot for each endothelial marker.


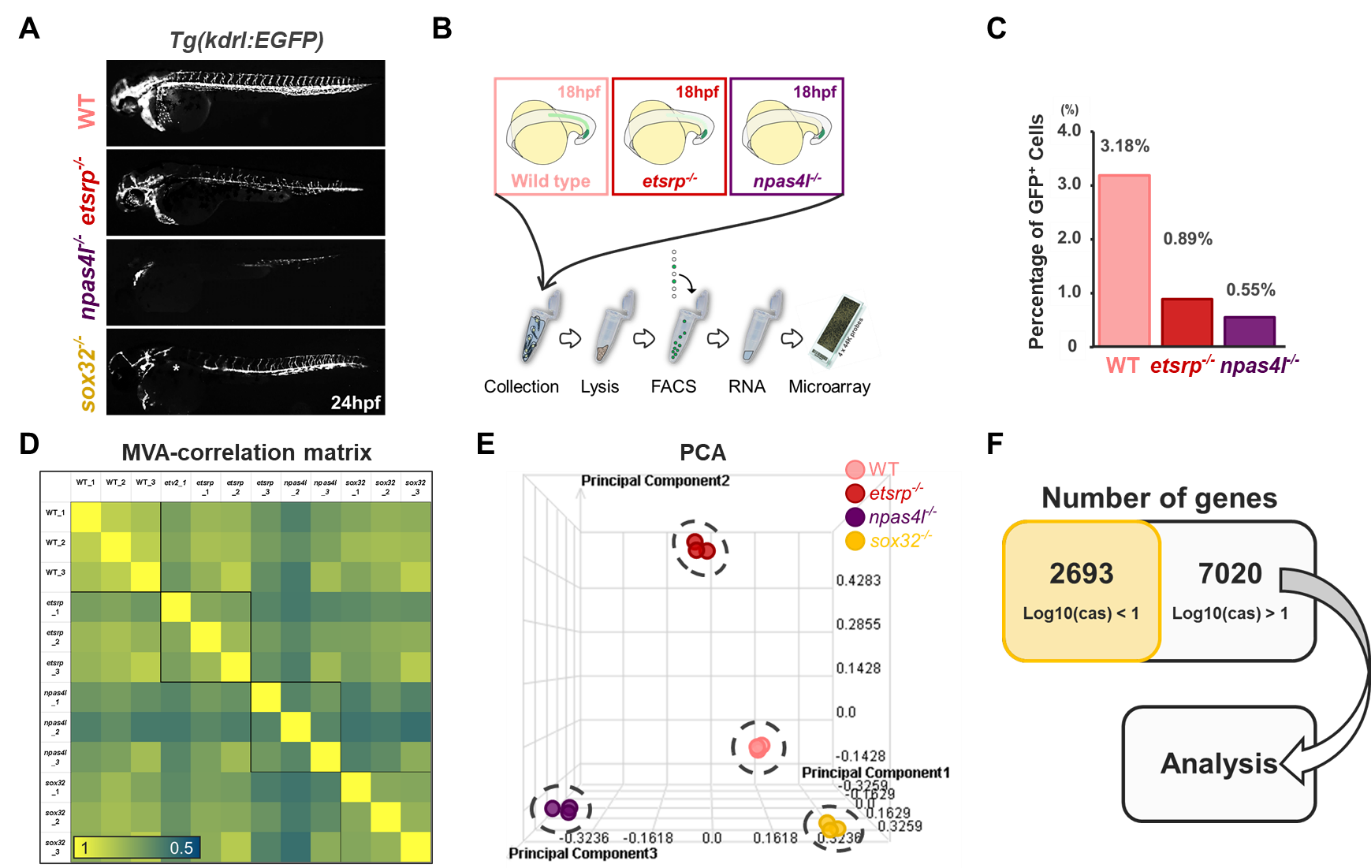


**Supplementary Figure 3: Workflow of microarray using avascular ECs**

(**A**) Fluorescence micrographs of 24hpf wild-type, *etsrp*^-/-^, *npas4l*^-/-^, and *sox32*^-/-^ show distinct vascular phenotypes. (**B**) Schematic diagram for isolation of endothelial cells. Single cell suspension from18hpf embryos with distinct genotype in *Tg(kdrl:eGFP)* background were sorted for eGFP expression. (**C**) At 18hpf, *etsrp*^-/-^ and *npas4l*^-/-^ contain significantly less endothelial cells compared to wild-type. (**D**) Correlation matrix of transcriptomes shows close relationship among sets with the same genotype. (**E**) Principal component analysis clusters experimental sets with the same genotype. (**F**) Subtraction of the transcripts of which expression is downregulated in *sox32*^-/-^ leaves 7020 transcripts for further analyses.


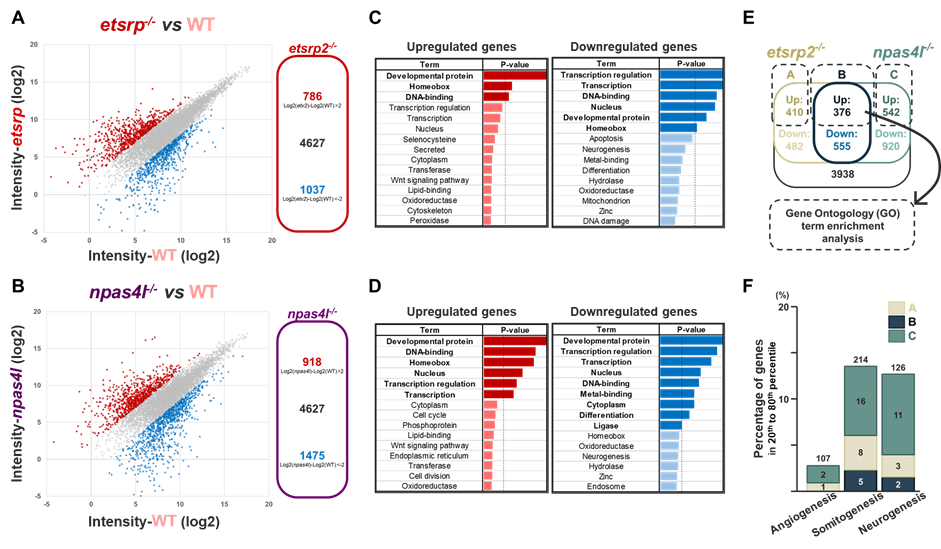


**Supplementary Figure 4: Transcriptomic signatures of *etsrp*^-/-^ and *npas4l*^-/-^ ECs**

(**A-B**) Differentially expressed genes (DEG) between the *kdrl:*eGFP^+^ cells isolated from wild-type and *etsrp^-/-^* (**A**) or *npas4l^-/-^* (**B**) embryos were analyzed. (**C-D**) Gene ontology analysis of transcripts enriched in *etsrp*^-/-^ (**C**) and *npas4l*^-/-^ (**D**) endothelial cells. Transcript associated with developmental proteins, homeobox, DNA binding domains are most abundant in both avascular endothelial cells. (**E**) Transcripts of which expression was altered in both avascular endothelial cells, 376 upregulated and 555 downregulated, were selected for further analyses. (**F**) Transcripts enriched in both avascular endothelial cells were significantly more prevalent in transcripts associated with somitogenesis or neurogenesis than angiogenesis.


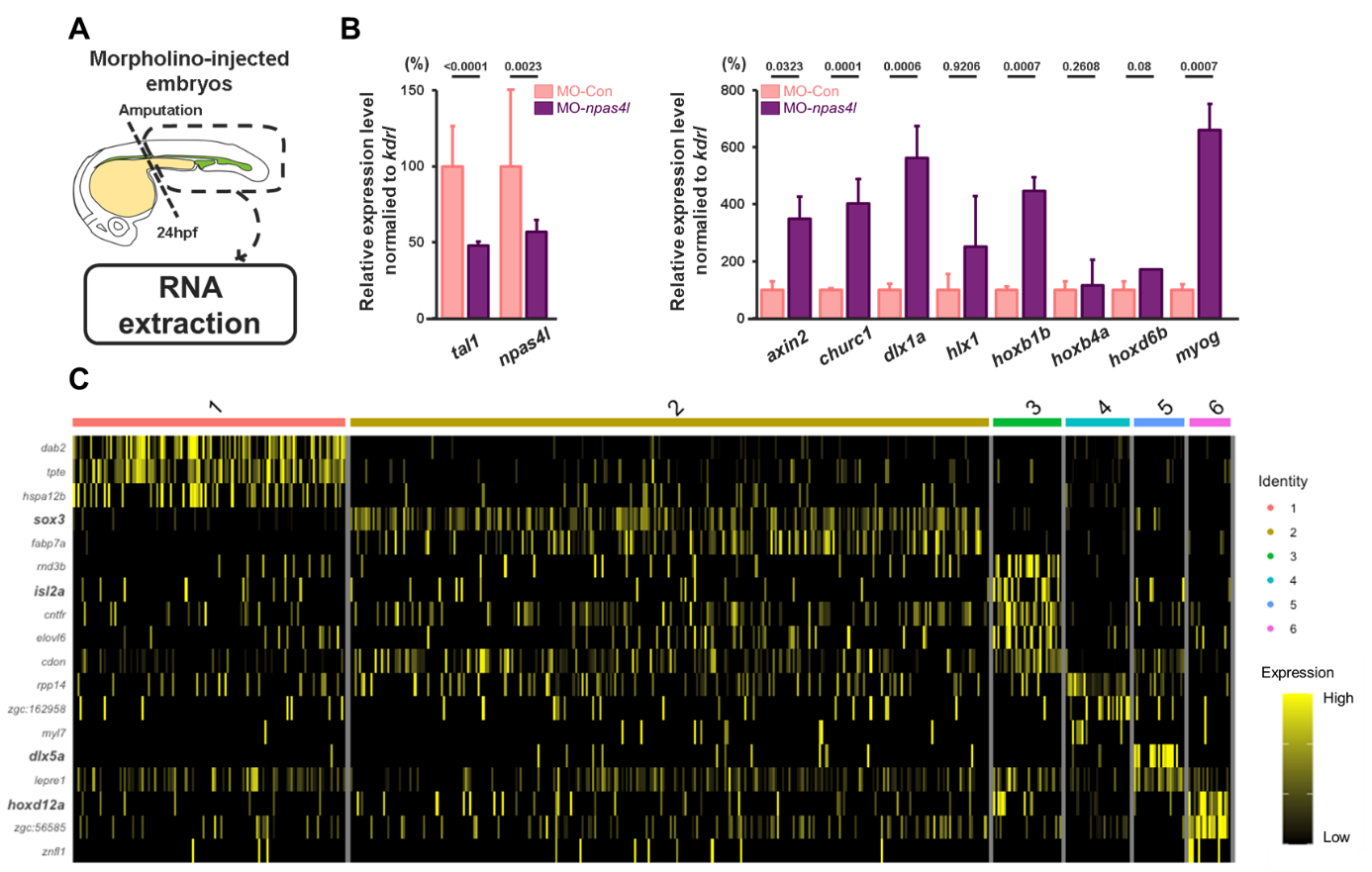


**Supplementary Figure 5: Validation of transcriptomic analyses**

(**A**) Schematic drawing of RNA extraction process from *npas4l*-MO injected embryos. Area posterior to the yolk extension were isolated for further experiments. (**B**) qRT-PCR analyses were normalized to *kdrl* expression. *tal1* and *npas4l* were downregulated in *npas4l* MO-injected embryos. In contrast, *axin2*, *churc1*, *dlx1a*, *hlx1*, *hoxb1b*, *hoxb4a*, *hoxd6b*, and *myog* transcripts are elevated in *npas4l* MO-injected embryos. (**C**) Three transcripts for each cluster are shown in the heat map.

**Supplementary Movies**

**Supplementary Movie 1: Emergence of *kdrl*:eGFP^+^ cells in *etsrp*^-/-^ and *npas4l*^-/-^**

The posterior region of *etsrp*^-/-^ and *npas4l*^-/-^ was imaged between 20hpf to 24hpf, along with the wild-type sibling. Asterisks point newly formed eGFP^+^ endothelial cells in avascular mutants

**Supplementary Movie 2: Formation of intersegmental vessels in *npas4l*^-/-^ between 40hpf and 44hpf**

The trunk region of *npas4l*^-/-^ was imaged between 40hpf to 44hpf, along with the wild-type sibling. Asterisks point the intersegmental vessels in *npas4l*^-/-^.

**Supplementary Tables**

**Supplementary Table 1: List of primers used in the experiments**

**Supplementary Table 2: Representative list of genes of which expression was altered in ECs isolated from avascular mutants**

**Supplementary Table 3: Representative list of cluster specific transcripts**
